# Esophageal dedifferentiated liposarcoma resected by the cervical approach: a case report

**DOI:** 10.1186/s40792-024-01990-y

**Published:** 2024-08-28

**Authors:** Kazuki Omachi, Keisuke Kosumi, Takumi Tanizaki, Tasuku Toihata, Masaaki Iwatsuki, Yoshifumi Baba, Yuji Miyamoto, Naoya Yoshida, Hideo Baba

**Affiliations:** Department of Gastroenterological Surgery, Graduate School of Medical Sciences, Kumamoto University, 1-1-1 Honjo, Chuo-Ku, Kumamoto, 860-8556 Japan

**Keywords:** Esophagus, Dedifferentiated liposarcoma, Cervical approach

## Abstract

**Background:**

While liposarcomas tend to mainly occur in the soft tissues of the extremities and retroperitoneum, esophageal liposarcoma is rare. Herein, we report a case of a patient who underwent complete resection of an esophageal dedifferentiated liposarcoma via the cervical approach, leading to the preservation of the esophagus.

**Case presentation:**

A 69-year-old man underwent an upper gastrointestinal endoscopy, as a result of which a submucosal-like tumor was observed. Upper gastrointestinal imaging showed a 12-cm tumor with a stalk arising from the esophageal entrance, extending to the middle intrathoracic esophagus, with a normal surface mucosa. Endoscopic ultrasound-fine needle aspiration biopsy showed that the nuclei of tumors cells were positive for murine double minute (MDM) and weakly positive for cyclin-dependent kinase 4 (CDK4). We diagnosed the tumor as the esophageal dedifferentiated liposarcoma, and planed tumor resection via the cervical approach. The tumor was successfully resected and the postoperative course was uneventful.

**Conclusion:**

This case report highlights the use of tumor resection via the cervical approach as a good option for esophageal liposarcoma.

## Introduction

Liposarcomas account for 15–25% of soft tissue malignancies, occurring mainly in the soft tissues of the extremities and retroperitoneum [1]. Among gastrointestinal mesenchymal tumors occurring in the esophagus, leiomyoma is the most common, while liposarcomas are less frequent. In fact, esophageal liposarcomas only account for 1.2–1.5% of all gastrointestinal liposarcomas [2]. The first case of a primary esophageal liposarcoma was reported in 1983, with 68 cases having been reported since [3, 4]. Esophageal dedifferentiated liposarcoma is rare among esophageal liposarcomas, with a reported rate of 9% among esophageal liposarcomas [5]. This condition is difficult to diagnose until the appearance of symptoms, which include dysphagia, respiratory symptoms, and vomiting of the polypoid lesion through the mouth [6]. Symptomatic cases require surgical resection. Herein, we report a case of a patient who underwent complete resection of an esophageal dedifferentiated liposarcoma via the cervical approach, leading to the preservation of the patient’s esophagus.

## Case presentation

**Fig. 1:**
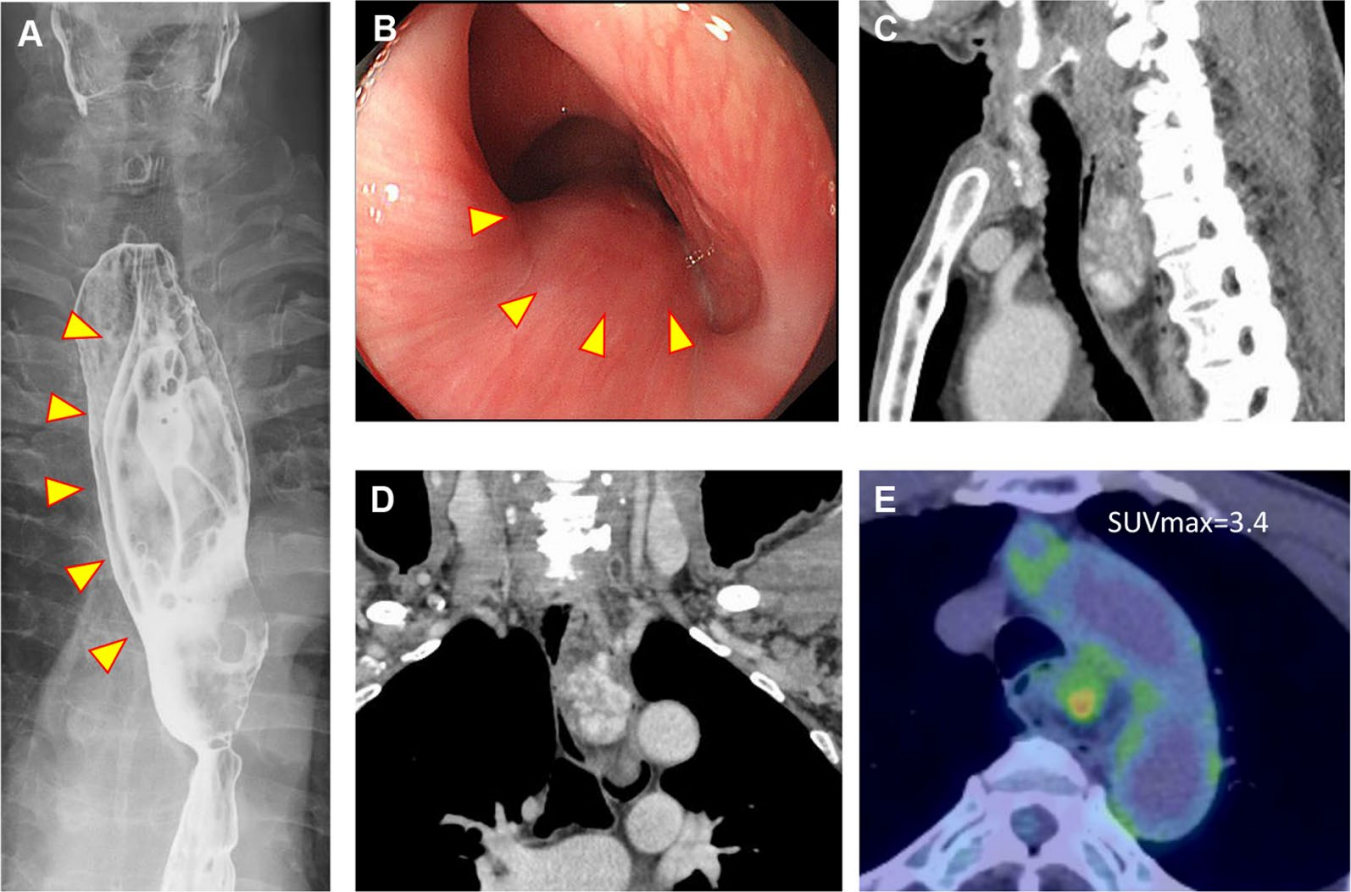
Preoperative X-ray fluoroscopy, upper gastrointestinal endoscopy, and PET–CT. **A** The tumor extends from the cervical esophagus to the upper thoracic esophagus. **B** Tumor stalk arose from the esophageal entrance. **C**, **D** The tumor was located from neck to the middle intrathoracic esophagus. The inside was a mixture of rich components with a potentiating effect and fatty components. **E** A low level of FDG accumulated (SUV max = 3.4) at the part of the enrichment of the tumor. There were no findings suggesting the invasion of tumor cells to neighboring organs

A 69-year-old man presented to a clinic for an esophageal abnormality pointed out during a prior medical examination. He underwent an upper gastrointestinal endoscopy, during which a submucosal-like tumor was observed. He was subsequently referred to our hospital for treatment, at which time he admitted discomfort in the cervical region. He had a history of cataract, enlarged prostate, and right inguinal hernia. His tumor markers were not found to be increased (carcinoembryonic antigen: 2.1 ng/mL, squamous cell cancer antigen: 1.0 ng/mL). Upper gastrointestinal imaging showed a 12-cm tumor with a stalk arising from the esophageal entrance, extending to the middle intrathoracic esophagus, with a normal surface mucosa (Fig. 1A, B). Contrast-enhanced computed tomography (CT) showed a tumor from the neck to the middle intrathoracic esophagus, the inside of which was comprised of a mixture of rich components with a potentiating effect and fatty components (Fig. 1C, D). Positron emission tomography–CT (PET–CT) showed a low level of FDG accumulation (SUV max = 3.4) in the region associated with tumor enrichment (Fig. 1E). There were no findings suggesting invasion of tumor cells to neighboring organs, lymphadenopathy, or distant metastasis. The patient then underwent endoscopic ultrasound-fine needle aspiration biopsy, which showed that the tumor was composed of mature adipocytes and lipoblasts with large irregular hyperchromatic nuclei and a mix of poorly differentiated adipocytes. Immunobiologically, the nuclei of the tumor cells were positive for murine double minute (MDM), weakly positive for cyclin-dependent kinase 4 (CDK4) and smooth muscle actin (SMA), and negative for KIT, CD34, S100, and Desmin. Thereafter, the patient was diagnosed with esophageal dedifferentiated liposarcoma.

**Fig. 2:**
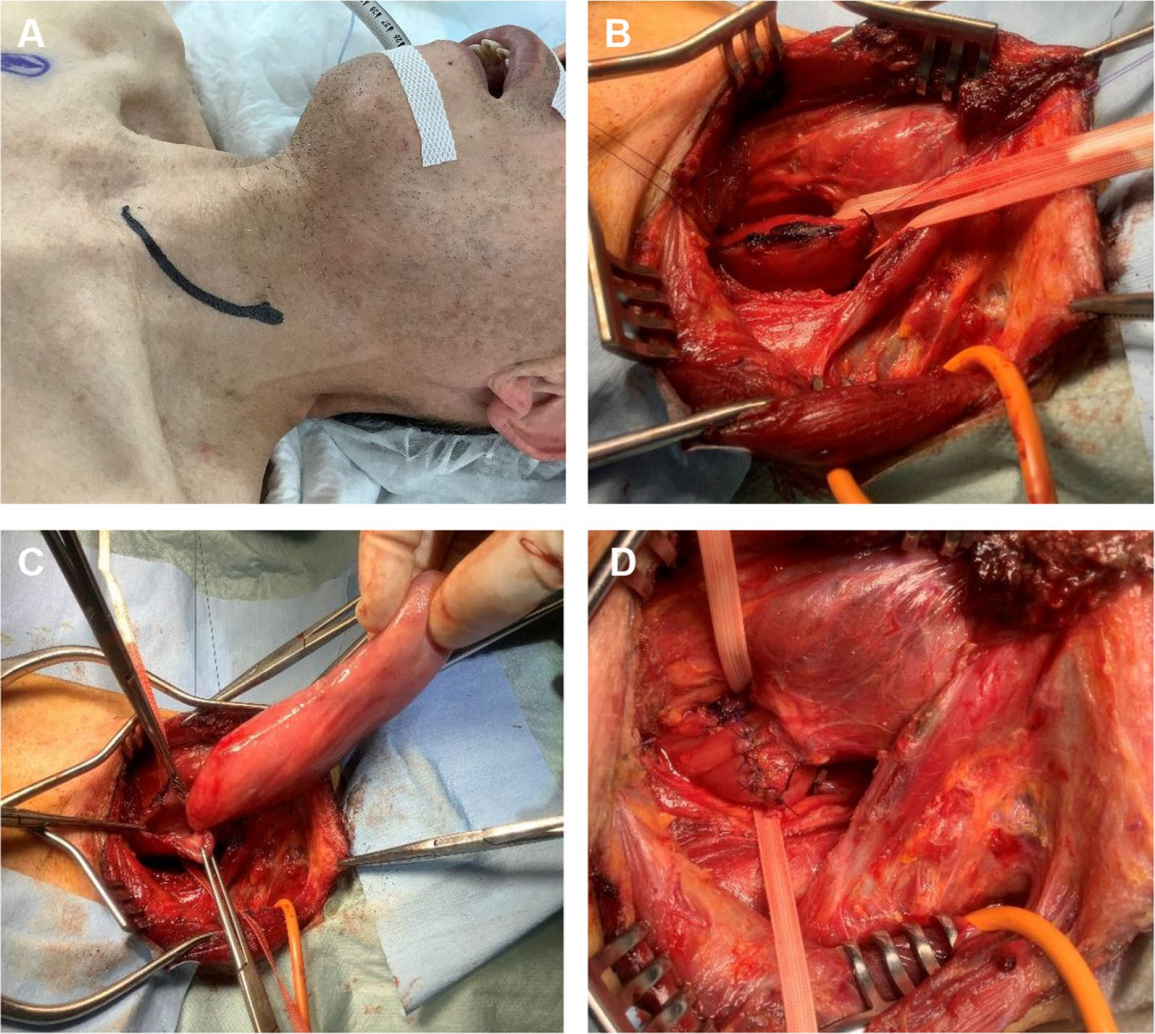
Operative findings. **A** We made a skin incision on the left side of the neck. **B** We directly made an incision on the ventral side of the cervical esophagus. **C** The tumor was pulled out from the esophageal lumen and resected from esophageal wall. **D** The mucous and muscle layers of the esophagus were closed with nodular sutures

Based on these findings, we decided to attempt tumor resection via the cervical approach, a minimally invasive procedure without lymph node dissection. After placing the patient under general anesthesia, we made a skin incision on the left side of the neck (Fig. 2A), and cut the lateral side of the anterior cervical muscles. We preserved the sternocleidomastoid muscle, and entered the inside of the common carotid artery. Next, we reached the wall of the esophagus, and taped it (Fig. 2B). We made an incision directly on the esophageal wall and pulled out the tumor from the esophageal lumen (Fig. 2C). We transected the stalk of the tumor from esophageal wall, and extracted it. Intraoperative rapid pathological examination confirmed that there were no malignant findings at the resected margin. The mucous and muscle layers of the esophagus were closed with nodular sutures (Fig. 2D). The duration of the surgery was 3 h and 56 min, and blood loss was 60 mL. The patient had an uneventful postoperative course without any complications, and was discharged at 17 days after the operation. He had no recurrence for twelve months after resection.

**Fig. 3:**
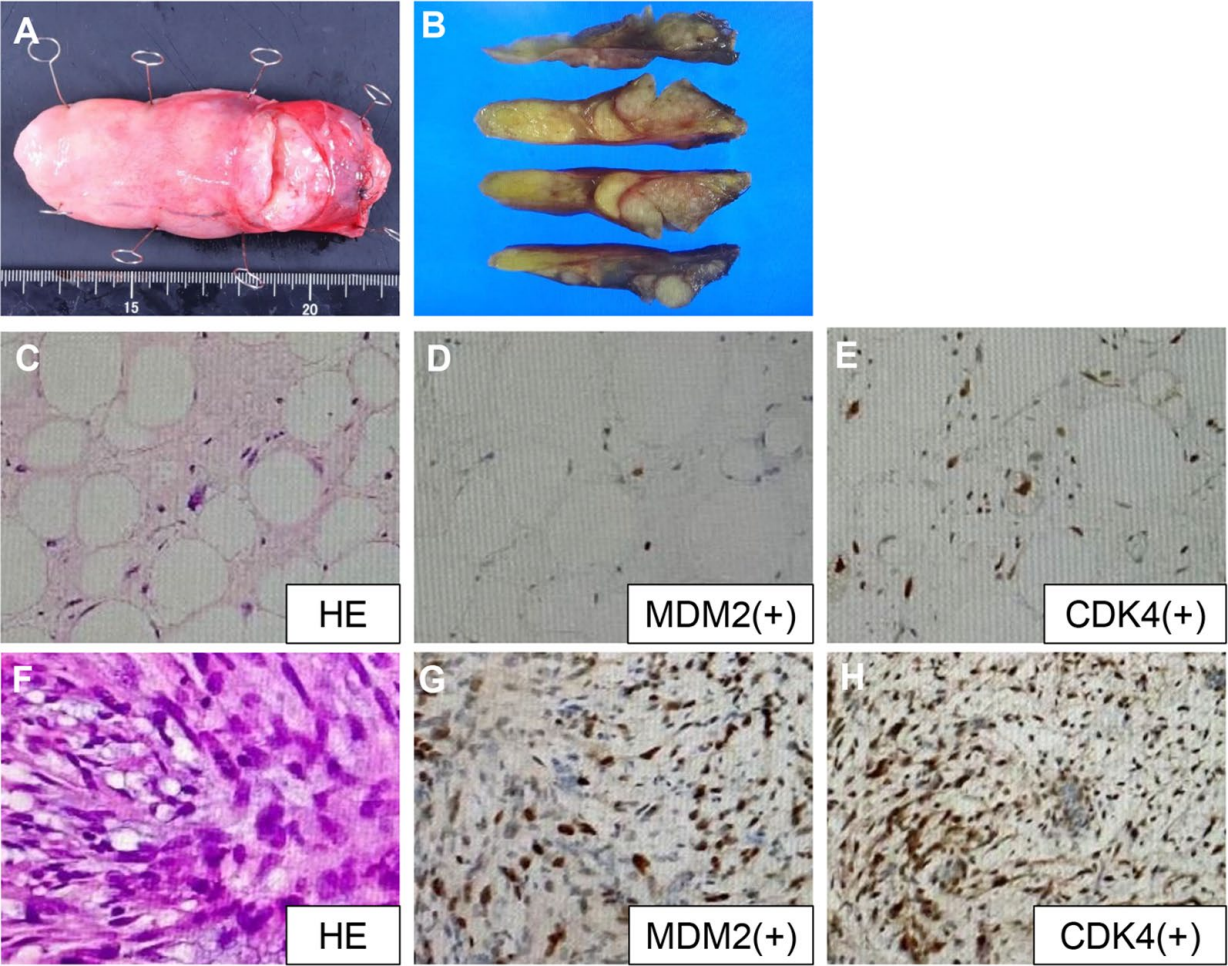
The surgical specimen and histopathological examination. Histopathological examination showed that the tumor was composed of well-differentiated and dedifferentiated liposarcoma. **A** The excised specimen was 9.5 × 4.0 × 2.3 cm in size. **B** The tumor cross section showed that the tumor contained adipose and elastic tissue. **C** The tumor was composed of mature adipocytes and lipoblasts with large irregular hyperchromatic nuclei. **F** The tumor was mixed with poorly differentiated adipocytes. **D**, **E**, **G**, **H** The nuclei of the tumors cells were positive for murine double minute (MDM) and weakly positive for cyclin-dependent kinase 4 (CDK4)

The excised specimen was 9.5 × 4.0 × 2.3 cm in size (Fig. 3A, B). Histological examination showed that the tumors were a mixture of mature and poorly differentiated adipocytes. Immunohistologically, the nuclei of tumor cells were positive for MDM2 and CDK4 (Fig. 3C–H).

## Discussion

Liposarcoma is the most common malignant soft tissue tumor in adults and usually occurs in the extremities and retroperitoneum [1, 7]. By contrast, primary gastrointestinal liposarcomas are much rarer, with a reported incidence at autopsy between 0.1 and 5.8% [8, 9]. Esophageal liposarcoma is extremely rare, accounting for 1.2‒1.5% of all gastrointestinal liposarcomas [2]. Esophageal liposarcoma was reported for the first time in 1983 [3]. To the best of our knowledge, there have been only 68 cases of esophageal liposarcomas in 54 reports in the literature since then [4, 10, 11]. Liposarcoma is generally a slowly growing tumor, usually growing to a considerable size that causes symptoms, such as dysphagia and weight loss [10]. In this case, the patients only had cervical discomfort and no difficulty eating or weight loss.

Liposarcoma is histologically classified into four types: well-differentiated, dedifferentiated, myxoid, and pleomorphic. The most common subtype of esophageal liposarcoma is the well-differentiated type, which is characterized histologically by adipocytes containing atypical nuclei and atypical stromal cells with increased nuclear chromatin condensation [10]. Immunostaining for MDM2 and CDK4 is useful for the diagnosis of liposarcoma. The expression of MDM2 and CDK4 genes in chromosome 12q13-15 was found to be amplified in both well-differentiated and dedifferentiated liposarcoma, and immunostaining showed high sensitivity and specificity in both tumors, which can be used for differential diagnosis. MDM2 and CDK4 immunostaining is a helpful adjunct to distinguish dedifferentiated liposarcoma from poorly differentiated sarcomas [12].

Tumor resection is the only curative treatment for esophageal liposarcoma [13, 14]. Although surgical procedures for the treatment of this type of sarcoma were more common in the past, in recent years, endoscopic resections have also often been performed. If the tumor is relatively small, pedunculated, has a single stalk, and with no large vessels in the stalk, endoscopic resection becomes an option for treatment [15]. In the case of endoscopic resection, when the tumor is too large for picking out orally, it must be removed through a cervical incision [16]. Therefore, endoscopic resection is considered useful for tumors that can be removed orally.

Esophagostomy could be considered a feasible procedure when the tumor is too large to resect with an endoscope or has large vessels in the stalk. The resection of tumors that develop at the cervical esophagus via the cervical approach is a minimally invasive surgical technique [10]. There have been seven previous reports of cases of esophageal liposarcoma that were completely resected and removed using only a cervical incisional approach (Table 1) [3, 10, 11, 17–20]. This was the first case of esophageal dedifferentiated liposarcoma resected by the cervical approach alone. However, resection via the cervical approach has several limitations. For resection via the cervical approach, the tumor stalk must be located in the cervical esophagus to ensure there is no chance of airway obstruction when a tumor is pulled out, as well as that there is no need for lymph node dissection [11]. Therefore, it is important to accurately evaluate the features of the tumor, including its location, size, and the histological type, by preoperative imaging and biopsy. In the present case, we confirmed that the stalk of the tumor was at the cervical esophagus using upper gastrointestinal endoscopy. Endoscopic ultrasound-fine needle aspiration made it possible to diagnose the tumor as dedifferentiated liposarcoma before surgery. While well-differentiated liposarcoma have no metastatic potential, dedifferentiated liposarcoma carry a poorer prognosis and behave as a high-grade sarcoma capable of metastasizing to distant sites, including the lungs, liver, bone, skin and soft tissue, or brain [21, 22]. In this context, an evaluation of lymph node and distant metastasis is important for determining the course of treatment. In our case, contrast-enhanced CT and PET–CT revealed that there were no lymph node or distant metastases. As such, we judged that the tumor was completely resectable. The previous cases of esophageal dedifferentiated liposarcoma were treated by endoscopic or thoracoscopic resection or thoracotomy of the tumor (Table 2) [23–29]. In a previous case, the tumor was too large to be pulled out of the esophagus and could not be moved out past the upper esophageal sphincter. Therefore, it was pushed back into the stomach, cut into smaller pieces using a hot snare, and removed in a piecemeal fashion [28]. In other case, the tumor extending outside the esophagus was removed thoracoscopically [29]. In this case, endoscopic manipulation was difficult because the tumor was in the neck. In addition, the tumor was too large to be removed endoscopically. We therefore planned to resect the tumor by the cervical approach. This highlights the fact that minimally invasive resection of liposarcoma requires an appropriate degree of preoperative examination.

**Table 1:** Literature review of eight cases, including our case, of esophageal liposarcoma that were removed only through the cervical approach

Author	Year of publication	Age sex	Type	Symptom	Cervical approach	Tumor size (cm)	Histology
Mansour [3]	1983	53 M	Polypoid	Dysphagia	Right	4 × 3	Myxoid
Salis [19]	1998	73 M	Polypoid	Dysphagia Vomiting	Left	15 × 6	Well-differentiated
Maruyama [20]	2007	50 M	Polypoid	Cough	Left	18.5 × 8.5 × 4	Well-differentiated
Saleh [18]	2013	62 M	Polypoid	Dysphagia Weight loss	Left	24	Well-differentiated
Dowli [17]	2014	64 M	Polypoid	Dysphagia	Left	15 × 7 × 3	Well-differentiated
Furukawa [10]	2021	72 F	Polypoid	Dysphagia	Left	15 × 7 × 5	Well-differentiated
Okura [11]	2021	66 M	Polypoid	Dysphagia	Left	23 × 8.5	Well-differentiated
Omachi	2023	69 M	Polypoid	Cervical discomfort	Left	9.5 × 4.0 × 2.3	Dedifferentiated

**Table 2:** Literature review of seven cases, including our case, of esophageal dedifferentiated liposarcoma that were removed

Author	Year of publication	Age sex	Type	Symptom	Treatment	Tumor size (cm)	Follow-up
Will [23]	2007	60 M	Polypoid	Dysphagia	Endoscopic resection	20 × 4	No recurrence at 12 months
Xu [24]	2008	50 M	Polypoid	Dysphagia	Endoscopic resection	11 × 5 × 5	No recurrence at 36 months
Torres-Mora [25]	2012	81 M	Polypoid	None	Endoscopic resection	7.3 × 2.8	N/A
Brett [26]	2016	75 M	Polypoid	Dysphagia	Endoscopic resection	5.0 × 2.8	No recurrence at 20 months
Weaver [27]	2018	73 M	Polypoid	Neck lump	Thoracotomy	30.5 × 16.5 × 8.5	No recurrence at 4 months
Parikh [28]	2019	58 M	Polypoid	Dysphagia	Endoscopic resection	18	N/A
Yi-Wang [29]	2020	38 F	Polypoid	Dysphagia	Thoracoscopic resection	2.1 × 1.5	No recurrence at 20 months
Omachi	2023	69 M	Polypoid	Cervical discomfort	cervical approach	9.5 × 4.0 × 2.3	No recurrence at 12 months

The prognosis of esophageal liposarcoma depends on the histological type and grade of the tumor, its location, and the status of the surgical margins [30]. Of all histologic types, well-differentiated liposarcoma has been reported to have the highest 5-year survival and the lowest recurrence rate [31]. Myxoid and pleomorphic liposarcomas have higher recurrence rates and worse prognosis than well-differentiated liposarcoma. A review of the literature showed combined 5- and 10-year survival probabilities of 51.5 and 34.8%, respectively, for all patients with primary dedifferentiated liposarcoma [32]. There are seven previous cases of esophageal dedifferentiated liposarcoma that were completely resected (Table 2) [23–29]. In all cases, the lymph nodes were not radically dissected, and there was no recurrence. Macroscopically complete surgical resection (R0/R1) is predictive of improved overall survival in patients with dedifferentiated liposarcoma. In our case, we were able to completely remove the tumor. As such, this may have a good therapeutic effect. However, follow-up with upper gastrointestinal endoscopy is necessary because of the potential for recurrence and the possibility of a new esophageal carcinoma in any remnants of the esophagus.

PET–CT is useful in malignant tumors. Previous reports have not described PET–CT findings in esophageal dedifferentiated liposarcoma. Although there have been previous findings of similarly low concentration in cases of differentiated liposarcoma, those reports did not discuss the PET–CT values [10]. In this case, PET–CT showed a low level of FDG accumulation in the region associated with tumor enrichment. Cases need to be accumulated for consideration of PET–CT values.

## Conclusion

In this paper we report a case of esophageal dedifferentiated liposarcoma resected using the cervical approach. Our observations and results indicate that preoperative endoscopy and biopsy were helpful for determining the surgical site and process. Taken together, the cervical approach was found to be useful when the tumor stalk was in the cervical esophagus.

## Data Availability

All data supporting the conclusions of this article are included within the published article and the accompanying images.

## Abbreviations

CT: Computed tomography
PET–CT: Positron emission tomography–CT
MDM: Murine double minute
CDK4: Cyclin-dependent kinase 4
SMA: Smooth muscle actin
